# Knowledge, Awareness, and Self-Reported Association Between Anxiety Symptoms and Gastroesophageal Reflux Disease in Jeddah, Saudi Arabia: A Cross-Sectional Study

**DOI:** 10.7759/cureus.114963

**Published:** 2026-08-22

**Authors:** Ekram N Abdalhaleem, Renad H Alzahrani, Amjaad M Alzharani, Lama M Alzahrani, Ibtehal M Alqaozi, Ebtihal A Alqaozi, Bashir A Yousef, Mohamed A El-Moselhy

**Affiliations:** 1 Clinical Pharmacy and Pharmacology, Ibn Sina National College for Medical Studies, Jeddah, SAU; 2 Pharmacology and Toxicology, Al-Azhar University, Cairo, EGY; 3 Medicine, Ibn Sina National College for Medical Studies, Jeddah, SAU; 4 Pharmacology, University of Khartoum, Khartoum, SDN; 5 Pharmacology and Toxicology, Minia University, Minya, EGY

**Keywords:** anxiety, cross-sectional study, factors, gastroesophageal reflux disease, gerd, jeddah, knowledge, public awareness, saudi arabia

## Abstract

Background: Gastroesophageal reflux disease (GERD) is a common gastrointestinal disorder characterized by the reflux of gastric contents into the esophagus, causing symptoms such as heartburn, acid regurgitation, and extraesophageal manifestations. Psychological stress may worsen reflux symptoms by altering gastrointestinal motility, increasing esophageal sensitivity, and disrupting the brain-gut axis, while persistent reflux can negatively affect quality of life and contribute to psychological distress. Despite this evidence, public understanding of this relationship remains limited, particularly in Saudi Arabia.

Aim: This study aimed to assess the self-reported relationship between anxiety symptoms and GERD-related symptoms, evaluate public awareness regarding this relationship, and identify associated sociodemographic and lifestyle factors among participants in Jeddah, Saudi Arabia.

Methods: A cross-sectional study was conducted among adults residing in Jeddah using a convenience nonprobability sampling technique. The minimum required sample size was calculated using G*Power software (ver. 3.1 Heinrich-Heine-Universität Düsseldorf, Düsseldorf, Germany; α = 0.05, power = 95%, effect size = 0.30, and five degrees of freedom), yielding a minimum of 220 participants. Data were collected through a bilingual (Arabic and English) self-administered questionnaire developed by the research team and reviewed by experts for face and content validity, as well as an electronic questionnaire distributed via social media. The survey assessed sociodemographic characteristics, lifestyle habits, reflux symptoms, anxiety-related factors, and knowledge of the self-reported association between anxiety symptoms and GERD. Data were analyzed using IBM Statistical Package for the Social Sciences, version 28.0 (IBM Corp., Armonk, NY; 2021). Descriptive statistics and chi-square tests were applied, with statistical significance set at p < 0.05.

Results: A total of 388 participants were included in the study. Of these, 90 (23.2%) reported having been diagnosed with GERD by a healthcare professional, while 167 (43.0%) describe the proportion of participants who reported regularly experiencing anxiety symptoms. Heartburn was the most frequently reported symptom, with 226 cases (58.2%). A significant self-reported association was observed between GERD and age (p = 0.027), as well as between anxiety symptoms and several sociodemographic and lifestyle factors (p < 0.05). Participants demonstrated variable levels of knowledge regarding the relationship between anxiety and GERD.

Conclusion: The study found that self-reported anxiety symptoms and reflux-related symptoms were commonly reported among participants in Jeddah. A substantial proportion of participants also perceived worsening of reflux-related symptoms during periods of anxiety or stress. These findings reflect self-reported symptoms and perceptions and do not establish a clinical diagnosis of anxiety or anxiety-associated GERD or a causal relationship between the two conditions.

## Introduction

Gastroesophageal reflux disease (GERD) is a common gastrointestinal disorder characterized by the reflux of gastric and duodenal contents into the esophagus, resulting in symptoms such as heartburn, acid regurgitation, and various extraesophageal manifestations, including chronic cough, hiccups, abdominal bloating, and dysphagia [1]. Accumulating evidence indicates a bidirectional relationship between GERD and anxiety. Persistent reflux symptoms may increase psychological distress, whereas anxiety can increase GERD symptoms by altering gastrointestinal motility, increasing esophageal hypersensitivity, and disrupting the brain-gut axis through neuroendocrine mechanisms [2-5]. These interactions may worsen symptom severity, reduce treatment response, and negatively affect patients' quality of life [2,3,6].

Recent evidence suggests that chronic psychological stress may increase transient lower esophageal sphincter relaxations, delay gastric emptying, and enhance visceral hypersensitivity, thereby contributing to both the onset and persistence of GERD symptoms [5,6]. Conversely, persistent reflux symptoms, sleep disturbance, and chronic discomfort may increase emotional distress and predispose patients to anxiety and depressive symptoms, further reinforcing the bidirectional relationship between gastrointestinal and psychological disorders [2,3].

Despite growing recognition of this association, anxiety frequently remains underdiagnosed in patients with GERD because clinical attention is often directed toward gastrointestinal symptoms rather than accompanying psychological conditions. Consequently, many patients do not receive appropriate assessment or management for anxiety, which may contribute to persistent symptoms and poorer clinical outcomes [7]. It is one of the most dominant gastrointestinal disorders worldwide and imposes a substantial burden on patients' quality of life and healthcare systems. GERD affects approximately 13%-15% of the global population, although its frequency varies considerably across geographic regions because of differences in dietary habits, obesity rates, and lifestyle factors [8].

Several studies have demonstrated a significant association between GERD and anxiety [2,9,10]. However, public awareness of this relationship remains limited, particularly in the Middle East, where lifestyle and dietary patterns may influence the occurrence of GERD. Assessing community awareness and identifying factors associated with anxiety-related GERD are essential for improving early recognition, promoting preventive strategies, and supporting integrated approaches to patient care. The coexistence of GERD and anxiety has been associated with greater symptom severity, reduced health-related quality of life, increased healthcare utilization, and lower treatment satisfaction compared with patients without psychological comorbidities [11].

Although numerous clinical studies have examined the association between GERD and anxiety [2,9,10], relatively few have investigated public awareness of this relationship or evaluated knowledge regarding the self-reported association between anxiety symptoms and GERD within the general population, particularly in Saudi Arabia. This knowledge gap highlights the need for community-based research to support targeted educational and preventive strategies.

Aim and objectives

The present study aimed to evaluate the proportion of self-reported association between anxiety symptoms and GERD, assess the level of public knowledge and awareness regarding the relationship between anxiety and GERD, identify factors associated with this relationship, and evaluate the impact of GERD on lifestyle among adults in Jeddah, Saudi Arabia.

## Materials and methods

Study design and setting

A cross-sectional study was conducted among participants residing in Saudi Arabia.

Eligibility criteria

The study included participants residing in Saudi Arabia who were able to understand and complete the Arabic or English version of the electronic questionnaire and who provided electronic informed consent. The questionnaire collected information on anxiety symptoms, previous professional diagnosis of GERD, and reflux-related symptoms. Although the study was primarily focused on the adult population, 10 participants younger than 18 years (2.6% of the total sample) were included in the final dataset and were retained in the analysis. Their small representation of the study population was taken into consideration when interpreting age-related findings.

Sample size and sampling technique

A convenience nonprobability sampling technique was employed. The minimum required sample size was calculated using the G*Power software (Ver. 3.1 Heinrich-Heine-Universität Düsseldorf, Düsseldorf, Germany) with a significance level (α) of 0.05, a statistical power of 95% (1 - β = 0.95), an effect size of 0.30, and five degrees of freedom. The calculated minimum required sample size was 220 participants, and 388 participants were included in the final analysis.

Data collection and study instrument

Data were collected using a bilingual (Arabic and English) self-administered electronic questionnaire developed by the research team following an extensive review of the published literature on GERD, anxiety, and public awareness of the relationship between these conditions. The questionnaire was specifically designed to address the objectives of the present study and consisted of six sections covering 1) sociodemographic characteristics, 2) self-reported anxiety symptoms, 3) self-reported previous professional diagnosis of GERD, 4) self-reported reflux-related symptoms among participants, 5) the impact of GERD on lifestyle, and 6) knowledge and management of GERD-related anxiety. The questionnaire included dichotomous (Yes/No), multiple-choice, multiple-response, and five-point Likert-scale questions. It was distributed electronically using Google Forms (Google LLC, Mountain View, CA) through social media platforms (WhatsApp (Meta Platforms, Inc., Menlo Park, CA), X (X Corp., Bastrop, TX), Telegram (Telegram FZ-LLC, Dubai, United Arab Emirates), and Facebook (Meta Platforms, Inc.)), and participation was voluntary and anonymous. A pilot study was conducted among 20 participants to assess the clarity and comprehensibility of the questionnaire; these participants were excluded from the final analysis. Before data collection, the questionnaire was reviewed by experts in pharmacology and clinical research to assess its face and content validity, and the wording was revised accordingly.

The questionnaire was self-administered, and participants completed it independently without interviewer assistance. Before accessing the questionnaire, all participants were provided with an information sheet describing the study objectives, and electronic informed consent was obtained. The complete questionnaire is provided in the Appendix.

The components of the questionnaire were distinguished as study-specific questions from any components derived from previously established measures. Where formal reliability testing was not performed, this is now explicitly acknowledged rather than implying that psychometric validation was performed. The questionnaire was used to collect self-reported information regarding anxiety symptoms, GERD-related symptoms, and knowledge/awareness and was not intended to establish a clinical diagnosis of anxiety or GERD.

Ethical considerations

Ethical approval was obtained from the Institutional Review Board of the College of Medicine, Ibn Sina National College. Participation in the study was voluntary, and informed electronic consent was obtained from all participants before completing the questionnaire. Participants were informed of their right to decline to participate or withdraw from the study at any time without consequences. The confidentiality and anonymity of all collected data were maintained throughout the study.

Statistical analysis

Data were checked for completeness, coded, and entered into the IBM Statistical Package for the Social Sciences, version 28.0 (IBM Corp., Armonk, NY; 2021). Descriptive statistics were used to summarize the study variables. Categorical variables were compared using the chi-square test. Multivariable logistic regression models were used to examine associations between selected demographic/clinical variables and the study outcomes. Reference categories were specified for categorical variables, and adjusted odds ratios (ORs) with 95% confidence intervals (CIs) were reported. Statistical significance was set at p < 0.05. Given the small number of participants younger than 18 years, age-related findings for this subgroup were interpreted with caution.

Data presentation

The study findings were presented using tables and appropriate descriptive statistical summaries.

## Results

Table 1 summarizes the sociodemographic characteristics of the study participants. Female participants constituted the majority of the sample, 307 (79.1%), while male participants accounted for 81 (20.9%). More than half of the participants, 206 (53.1%), were aged between 18 and 40 years, followed by those older than 40 years, 172 (44.3%), whereas only 10 (2.6%) were younger than 18 years. Regarding marital status, 245 (63.1%) were married, and 143 (36.9%) were single. Most participants had a university education, 262 (67.5%), followed by secondary education, 79 (20.4%), and postgraduate education, 44 (11.3%), while only 0.8% had primary school education.

**Table 1:** Assessment of sociodemographic characteristics (n = 388)

Variables		Frequency (Percentage)
Age	<18	10 (2.6%)
	18-40	206 (53.1%)
	>40	172 (44.3%)
Gender	Male	81 (20.9%)
	Female	307 (79.1%)
‏Marital status	Single	143 (36.9%)
	Married	245 (63.1%)
Educational level	Primary school	3 (0.8%)
	Secondary school	79 (20.4%)
	University	262 (67.5%)
	Postgraduate studies	44 (11.3%)

Table 2 presents the frequency of anxiety and GERD-related characteristics among the study participants. Overall, among the 388 participants, 167 (43.0%) reported regularly experiencing anxiety symptoms, whereas 221 (57.0%) did not. Regarding the frequency of anxiety symptoms, which was assessed separately among all participants, 42 (10.8%) reported never experiencing anxiety symptoms, 127 (32.7%) rarely, 94 (24.2%) weekly, 56 (14.4%) monthly, and 69 (17.8%) daily. Only 90 (23.2%) had been previously diagnosed with GERD by a healthcare professional, while 298 (76.8%) reported no formal diagnosis. Regarding GERD-related symptoms, heartburn was the most frequently reported symptom, 226 (58.2%), followed by a sour taste in the mouth, 110 (28.4%), other unspecified symptoms, 72 (18.6%), difficulty swallowing, 65 (16.8%), and chronic cough, 39 (10.1%). With respect to treatment, only 83 (21.4%) of participants reported using medication to relieve their symptoms, while the majority, 305 (78.6%), did not use any medication.

**Table 2:** Evaluation of the proportion of anxiety and GERD-related symptoms (n = 388) GERD: gastroesophageal reflux disease

Evaluation questions		Frequency (percentage)
Do you regularly experience symptoms of anxiety?	No	221 (57%)
	Yes	167 (43%)
Have you been officially diagnosed with GERD by a healthcare professional?	No	298 (76.8%)
	Yes	90 (23.2%)
What symptoms do you experience?		
Heartburn	No	162 (41.8%)
	Yes	226 (58.2%)
Sour taste in mouth	No	278 (71.6%)
	Yes	110 (28.4%)
Difficulty swallowing	No	323 (83.2%)
	Yes	65 (16.8%)
Chronic cough	No	349 (89.9%)
	Yes	39 (10.1%)
Other	No	316 (81.4%)
	Yes	72 (18.6%)
How often do you experience anxiety symptoms?	Never	42 (10.8%)
	Rarely	127 (32.7%)
	Daily	69 (17.8%)
	Weekly	94 (24.2%)
	Monthly	56 (14.4%)
Do you use any medication that improves GERD symptoms?	No	305 (78.6%)
	Yes	83 (21.4%)

Table 3 summarizes participants' awareness of the relationship between GERD and mental health. More than half of the participants, 216 (55.7%), reported having heard about the impact of GERD on mental health, whereas 172 (44.3%) had not. When asked whether GERD could affect mental health, 269 (69.3%) answered "Yes," 24 (6.2%) answered "No," and 95 (24.5%) were uncertain. Similarly, 222 (57.2%) of participants recognized that GERD may have consequences beyond physical symptoms, including psychological effects, whereas 43 (11.1%) were unaware of this association and 123 (31.7%) were unsure. Regarding sources of information about GERD, social media was the most frequently reported source, 123 (31.7%), followed by family, 71 (18.3%), healthcare professionals, 68 (17.5%), friends, 64 (16.5%), and personal research, 62 (16.0%).

**Table 3:** Assessment of public awareness regarding the relationship between GERD and mental health (n = 388) GERD: gastroesophageal reflux disease

Awareness questions		Frequency (percentage)
Have you heard about the impact of GERD on mental health?	No	172 (44.3%)
	Yes	216 (55.7%)
Do you think GERD can affect mental health?	No	24 (6.2%)
	Yes	269 (69.3%)
	I don't know	95 (24.5%)
Are you aware that GERD may have psychological as well as physical effects?	No	43 (11.1%)
	Yes	222 (57.2%)
	I don't know	123 (31.7%)
What is your main source of information about GERD?	Personal research	62 (16%)
	Family	71 (18.3%)
	Friends	64 (16.5%)
	Healthcare professionals	68 (17.5%)
	Social media	123 (31.7%)

Table 4 presents participants' perceptions of the self-reported association between anxiety symptoms and GERD. Nearly half of the participants, 180 (46.4%), reported that their GERD symptoms increased when they felt anxious or stressed, while 107 (27.6%) reported experiencing this association only occasionally, and 101 (26.0%) did not perceive such a relationship. When asked to describe the strength of the relationship based on their personal experience, 132 (34.0%) rated it as moderate, 86 (22.2%) as strong, and 26 (6.7%) as weak. In contrast, 9.3% reported no perceived association, whereas 108 (27.8%) were uncertain. Furthermore, 241 (62.1%) of participants indicated that they experienced periods during which anxiety appeared to exacerbate their GERD symptoms, while 147 (37.9%) did not report such a pattern.

**Table 4:** Determination of the self-reported association between anxiety symptoms and GERD (n = 388) GERD: gastroesophageal reflux disease

Association questions		Frequency (percentage)
Do you notice an increase in GERD symptoms when feeling anxious or stressed?	No	101 (26%)
	Yes	180 (46.4%)
	Sometimes	107 (27.6%)
How would you describe the connection between GERD and anxiety in your personal experience?	No connection	36 (9.3%)
	Weak	26 (6.7%)
	Moderate	132 (34%)
	Strong	86 (22.2%)
	Unsure	108 (27.8%)
Are there specific times when symptoms increase due to anxiety?	No	147 (37.9%)
	Yes	241 (62.1%)

Table 5 summarizes the impact of GERD on participants' lifestyle and daily activities. More than half of the participants, 212 (54.6%), reported that GERD negatively affected their overall quality of life, whereas 41 (10.6%) reported no effect and 135 (34.8%) did not notice any impact. Sleep disturbance was also commonly reported, with 223 (57.5%) indicating that GERD affected their sleep patterns, compared with 51 (13.1%) who reported no effect and 114 (29.4%) who were unaware of any impact. Dietary habits were the most affected aspect of lifestyle, with 276 (71.1%) participants reporting modifying their diet, such as avoiding specific foods, because of GERD. In contrast, only 29 (7.5%) reported no dietary impact, while 83 (21.4%) did not notice any changes. Regarding physical activity, only 87 (22.4%) participants reported engaging in regular exercise, whereas 301 (77.6%) did not exercise regularly. Furthermore, 175 (45.1%) reported that environmental factors, such as odors or noise, appeared to increase their GERD symptoms, while 213 (54.9%) did not perceive such an effect.

**Table 5:** Self-reported impact of reflux-related symptoms on lifestyle (n = 388) GERD: gastroesophageal reflux disease

Effects of GERD on lifestyle questions		Frequency (percentage)
How much does GERD impact your overall quality of life?	Affect	212 (54.6%)
	Doesn't affect	41 (10.6%)
	I didn’t notice	135 (34.8%)
Has GERD affected your sleep patterns?	Affect	223 (57.5%)
	Doesn't affect	51 (13.1%)
	I didn’t notice	114 (29.4%)
Has GERD influenced your diet? (e.g., avoiding specific foods)	Affect	276 (71.1%)
	Doesn't affect	29 (7.5%)
	I didn’t notice	83 (21.4%)
Do you exercise regularly?	No	301 (77.6%)
	Yes	87 (22.4%)
Do you notice that there are environmental factors (such as odors or noise) that affect your GERD?	No	213 (54.9%)
	Yes	175 (45.1%)

Table 6 presents participants' knowledge and approaches toward managing GERD-related anxiety. Regarding coping strategies, seeking medical advice was the most commonly reported approach, 179 (46.1%), followed by relaxation techniques, 170 (43.8%), and exercise, 161 (41.5%), while 30 (7.7%) reported using other methods. A considerable proportion of participants, 272 (70.1%), indicated that they had not received information from healthcare providers about strategies to reduce GERD-related anxiety, whereas only 116 (29.9%) had received such guidance. Regarding self-confidence in managing GERD-related anxiety symptoms, 199 (51.3%) reported a moderate level of confidence, while 66 (17.0%) reported very low confidence and another 66 (17.0%) reported low confidence. Only a minority of participants reported high, 44 (11.3%), or very high, 13 (3.4%), confidence levels. Notably, 302 (77.8%) of participants expressed interest in gaining further knowledge about the relationship between GERD and anxiety through educational materials or support programs, whereas 86 (22.2%) were not interested.

**Table 6:** Assessment of knowledge and management practices regarding GERD-related anxiety (n = 388) GERD: gastroesophageal reflux disease

Assessment questions	Frequency (percentage)
How do you manage anxiety related to gastroesophageal reflux?	
Exercise	161 (41.5%)
Relaxation techniques	170 (43.8%)
Medical consultation	179 (46.1%)
Other	30 (7.7%)
Have you received information from healthcare providers on ways to reduce GERD-related anxiety?	
No	272 (70.1%)
Yes	116 (29.9%)
On a scale of 1-5, how confident are you in managing anxiety symptoms related to GERD?	
Very low	66 (17%)
Low	66 (17%)
Middle	199 (51.3%)
High	44 (11.3%)
Very high	13 (3.4%)
Are you open to learning more about the link between GERD and anxiety through educational resources or support groups?	
No	86 (22.2%)
Yes	302 (77.8%)

Table 7 presents the bivariate analysis of the self-reported association between GERD and sociodemographic characteristics. Age was significantly associated with previous professional diagnosis of GERD (p = 0.027). Participants older than 40 years represented a higher proportion of those diagnosed with GERD, 51 (56.7%), compared with those without a diagnosis, 121 (40.6%). In contrast, participants aged 18-40 years accounted for a larger proportion of individuals without a GERD diagnosis, 169 (56.7%), than of those diagnosed, 37 (41.1%). Marital status was also significantly associated with GERD diagnosis (p = 0.042), with married participants representing a greater proportion of those diagnosed with GERD, 65 (72.2%), compared with those without a diagnosis, 180 (60.4%).

**Table 7:** Association between previous professional diagnosis of GERD and sociodemographic characteristics using Pearson chi-square test (n = 388) ^*^χ²: Pearson chi-square test. p < 0.05 is considered statistically significant GERD: gastroesophageal reflux disease

Variables		Diagnosed with GERD by a healthcare professional		χ² value	p value
		No	Yes		
Age	<18	8 (2.7%)	2 (2.2%)	7.249	0.027^*^
	18-40	169 (56.7%)	37 (41.1%)		
	>40	121 (40.6%)	51 (56.7%)		
Gender	Male	58 (19.5%)	23 (25.6%)	1.206	0.213
	Female	240 (80.5%)	67 (74.4%)		
‏Marital status	Single	118 (39.6%)	25 (27.8%)	4.5	0.042^*^
	Married	180 (60.4%)	65 (72.2%)		
Educational level	Primary school	3 (1%)	0 (0%)	2.934	0.402
	Secondary school	62 (20.8%)	17 (18.9%)		
	University	203 (68.1%)	59 (65.6%)		
	Postgraduate studies	30 (10.1%)	14 (15.6%)		

No statistically significant association was observed between gender and GERD diagnosis (p = 0.213), although male participants constituted a slightly higher proportion among participants diagnosed with GERD, 23 (25.6%), compared with those without a diagnosis (19.5%). Similarly, educational level was not significantly associated with GERD diagnosis (p = 0.402), despite a slightly higher proportion of participants with postgraduate education among those diagnosed, 14 (15.6%), compared with those without a diagnosis, 30 (10.1%).

Table 8 presents the results of the multivariate logistic regression analysis evaluating the self-reported association between sociodemographic characteristics and GERD diagnosis. Using participants younger than 18 years as the reference group, age was not significantly associated with GERD diagnosis after adjustment for other variables. Participants aged 18-40 years showed no significant association with GERD diagnosis (OR = 0.876, 95% CI: 0.18-4.29, p = 0.870), and those older than 40 years also demonstrated no statistically significant association (OR = 1.686, 95% CI: 0.35-8.22, p = 0.518). In contrast, marital status remained significantly associated with GERD diagnosis in the multivariate model. Married participants had approximately 1.7-fold higher odds of having a GERD diagnosis compared with single participants (OR = 1.70, 95% CI: 1.02-2.86, p = 0.043).

**Table 8:** Multivariable logistic regression analysis of sociodemographic factors associated with previous professional diagnosis of GERD (n = 388) ^*^Statistically significant OR: odds ratio; CI: confidence interval

Variables		OR	95% CI	p value
Age	<18	Reference	-	-
	18-40	0.876	0.18-4.29	0.87
	>40	1.686	0.35-8.22	0.518
‏Marital status	Single	Reference	-	-
	Married	1.7	1.02-2.86	0.043^*^

Table 9 presents the self-reported association between regularly experiencing anxiety symptoms and sociodemographic characteristics. Age was significantly associated with reported anxiety symptoms (p = 0.022). Participants younger than 18 years represented a higher proportion of those reporting anxiety symptoms, 8 (4.8%), compared with those without anxiety symptoms, 2 (0.9%). Gender was also significantly associated with anxiety symptoms (p = 0.025). Female participants constituted the most participants reporting anxiety symptoms, 141 (84.4%), compared with those without symptoms, 166 (75.1%), whereas male participants represented a lower proportion among participants with anxiety symptoms, 26 (15.6%), compared with those without symptoms, 55 (24.9%). In contrast, marital status was not significantly associated with anxiety symptoms (p = 0.344), although single participants represented a slightly higher proportion among those reporting anxiety symptoms, 66 (39.5%), compared with those without symptoms, 77 (34.8%). Similarly, educational level showed no statistically significant association with anxiety symptoms (p = 0.509), with comparable distributions across educational categories between participants with and without reported anxiety symptoms.

**Table 9:** Self-reported association between anxiety symptoms and sociodemographic characteristics using Pearson chi-square test (n = 388) ^*^χ²: Pearson chi-square test. p < 0.05 is considered statistically significant

Variables		Regularly experience symptoms of anxiety		χ² value	p value
		No	Yes		
Age	<18	2 (0.9%)	8 (4.8%)	7.644	0.022^*^
	18-40	12 6(57%)	80 (47.9%)		
	>40	93 (42.1%)	79 (47.3%)		
Gender	Male	55 (24.9%)	26 (15.6%)	4.452	0.025^*^
	Female	166 (75.1%)	141 (84.4%)		
‏Marital status	Single	77 (34.8%)	66 (39.5%)	0.705	0.344
	Married	144 (65.2%)	101 (60.5%)		
Educational level	Primary school	3 (1.4%)	0 (0%)	2.32	0.509
	Secondary school	44 (19.9%)	35 (21%)		
	University	149 (67.4%)	113 (67.7%)		
	Postgraduate studies	25 (11.3%)	19 (11.4%)		

Table 10 presents the results of the multivariate logistic regression analysis assessing the self-reported association between sociodemographic characteristics and reported anxiety symptoms. Using participants younger than 18 years as the reference group, those aged 18-40 years had significantly lower odds of reporting anxiety symptoms (OR = 0.159, 95% CI: 0.03-0.77, p = 0.022). Participants aged 18-40 years had lower odds of reporting anxiety symptoms than those younger than 18 years (OR = 0.159, 95% CI: 0.03-0.77, p = 0.022). Participants older than 40 years also had lower estimated odds, although this difference was not statistically significant (OR = 0.212, 95% CI: 0.04-1.03, p = 0.054). Given that only 10 participants (2.6%) were younger than 18 years, these age-related estimates should be interpreted with caution.

**Table 10:** Multivariate logistic regression analysis of sociodemographic characteristics self-reported association with anxiety symptoms (n = 388) ^*^Statistically significant OR: odds ratio; CI: confidence interval

Variables		OR	95% CI	p value	
Age	<18	Reference	-	-	
					18-40	0.159	0.03-0.77	0.022^*^	
	>40	0.212	0.04-1.03	0.054					
	Gender	Male	Reference	-	-	
						Female	1.8	1.07-3.02	0.027^*^	

## Discussion

The sociodemographic characteristics of the present study revealed a predominantly female sample, with most participants aged 18-40 years, married, and university educated. This demographic distribution is consistent with several recent studies investigating GERD and associated psychological conditions [2,9,10]. A 2025 study assessing the global burden of GERD among women of childbearing age reported significant sex differences, with women demonstrating higher age-standardized incidence, occurrence, and years lived with disability compared with men, which female-specific physiological and hormonal factors may influence [12].

Similarly, a 2024 population-based cross-sectional study from Taiwan involving a large cohort identified female sex and age between 40 and 59 years as important characteristics associated with GERD occurrence, reporting a community occurrence of approximately 25% [13]. Furthermore, a 2023 study conducted at Imam Mohammad Ibn Saud Islamic University in Saudi Arabia reported a higher proportion of GERD among female participants compared with male participants, supporting the female predominance observed in our study population [14].

In the present study, 43.0% of participants reported regularly experiencing anxiety symptoms, whereas others reported a previous diagnosis of GERD by healthcare professionals. Heartburn was the most frequently reported GERD-related symptom, followed by sour taste in the mouth and difficulty swallowing. These findings highlight the frequent coexistence of reflux-related symptoms and psychological complaints within the studied population. Additionally, a 2022 analysis published in the Journal of Neurogastroenterology and Motility reported that GERD incidence was significantly higher among individuals with mild anxiety and those with moderate-to-severe anxiety compared with individuals without anxiety, suggesting a possible dose-dependent association between anxiety severity and GERD occurrence [15].

However, a 2024 Mendelian randomization study provided a different perspective, suggesting that GERD may causally increase the probability of anxiety disorders. In contrast, the relation between anxiety and GERD was weaker in genetic analyses [16]. These findings indicate that although participants in our study perceived an association between anxiety and reflux symptoms, the underlying direction and mechanisms of this relationship remain complex.

The present study assessed participants’ awareness regarding the relationship between GERD and mental health. More than half of the participants reported that they had previously heard about the psychological impact of GERD, and others believed that GERD could affect mental health. However, social media was identified as the most common source of information, whereas healthcare professionals represented a few of the information sources. These findings indicate that although general awareness regarding the self-reported association between GERD and psychological health exists, important gaps remain in the depth and reliability of knowledge. A 2023 study reported that most participants had general knowledge about GERD; however, substantial gaps remained regarding its long-term complications, and unaware that GERD may contribute to serious outcomes such as dental erosion or cancer [17]. This supports our finding that awareness of GERD-related consequences may be incomplete despite the presence of general disease knowledge.

The AGA Clinical Practice Update published in 2022 emphasized the importance of providing standardized patient education regarding GERD mechanisms, lifestyle modifications, relaxation strategies, and the relationship between the gastrointestinal system and the brain-gut axis [18]. This recommendation highlights the need for structured educational approaches to improve patients’ understanding of the psychological aspects associated with GERD. Interestingly, our findings differed from those reported in a 2023 study, in which healthcare providers were the most common source of GERD information, followed by family members, whereas social media played a less prominent role [17]. The predominance of social media as an information source in our study may reflect changing patterns of health information-seeking behavior and emphasizes the importance of ensuring that online health information is accurate and evidence-based.

In this study, many participants reported increased GERD symptoms during periods of anxiety or stress, while an additional number occasionally experienced this self-reported association. Furthermore, some described the relationship between GERD symptoms and anxiety as moderate, and others considered it strong. These subjective findings are consistent with previous studies demonstrating an association between psychological distress and GERD severity. A 2022 study demonstrated that higher anxiety levels were associated with increased GERD occurrence, with moderate-to-severe anxiety showing significantly higher odds of GERD compared with individuals without anxiety [15].

Similarly, a 2022 systematic review and meta-analysis supported the association between GERD and psychological disorders, showing that individuals with GERD had a 2.57-fold higher occurrence of psychosocial disorders compared with individuals without GERD [18]. These findings support the interaction between gastrointestinal symptoms and psychological well-being. Nevertheless, a 2024 Mendelian randomization analysis suggested that although GERD may increase the probability of anxiety disorders, the evidence supporting anxiety as a direct causal factor for GERD was weaker, suggesting that the relationship may not be completely bidirectional at the genetic level [16]. Therefore, while our findings demonstrate a strong perceived self-reported association between anxiety and GERD symptoms, further longitudinal studies are needed to clarify causal pathways.

The present study demonstrated that GERD symptoms considerably affected participants’ daily lives. Dietary habits represented the most affected domain, with a high percentage reporting dietary modification, followed by sleep disturbances and negative effects on overall quality of life. Additionally, most participants reported not engaging in regular exercise. These findings are consistent with previous evidence demonstrating the impact of GERD on sleep and daily functioning. A 2023 prospective cohort study reported that GER symptoms were associated with an increased probability of poor sleep quality, particularly among women experiencing symptoms at least twice weekly, who showed a relatively low chance compared with those experiencing symptoms less than once monthly [19]. This supports our observation that more than half of participants experienced GERD-related sleep disturbances.

Furthermore, a 2024 meta-analysis confirmed a bidirectional association between GERD and sleep disorders, showing that insomnia increased the probability of GERD, while GERD was associated with poorer sleep quality [20]. In addition, a 2022 polysomnography-based study demonstrated objectively poorer sleep quality among GERD patients, with symptom severity influenced by sleeping position, particularly right-sided vs. left-sided sleep [21]. A 2024 study conducted in Bahrain reported that most GERD patients were married; however, the proportion reporting significant dietary modifications was lower than observed in our study, with only a few reporting dietary avoidance behaviors [22]. Differences in dietary patterns, cultural habits, and symptom perception may explain variations between populations. Moreover, an objective sleep study published in 2022 suggested that subjective sleep complaints among GERD patients may exceed objectively measured sleep impairment, highlighting the complex relationship between symptoms and perceived quality of life [21].

The present study assessed participants’ knowledge and approaches toward managing GERD-related anxiety. A considerable proportion of participants reported that they had not received information from healthcare providers regarding strategies to reduce GERD-related anxiety, despite a high percentage also expressing interest in obtaining further information through educational materials or support programs. The most reported management approaches were seeking medical advice, relaxation techniques, and exercise. These findings highlight the need for greater integration of psychological education into GERD management. A 2025 randomized controlled trial demonstrated that abdominal breathing exercises significantly improved GERD symptoms, enhanced quality-of-life scores, and reduced the need for on-demand proton pump inhibitor (PPI) use. The study suggested that breathing exercises may represent an alternative management approach with fewer potential adverse effects compared with continuous PPI therapy [23].

Similarly, a 2023 systematic review evaluating breathing exercises for GERD reported that diaphragmatic breathing training may reduce reflux symptoms by improving antireflux barrier function, particularly by increasing crural diaphragm tension [24]. These findings support the potential value of relaxation-based interventions as complementary strategies in GERD management. Furthermore, the American Gastroenterological Association’s 2022 Clinical Practice Update recommended that healthcare providers incorporate education regarding relaxation strategies and awareness of the brain-gut axis relationship as part of comprehensive GERD care [18]. This recommendation emphasizes that management should not be limited to acid suppression therapy but should also consider psychological and behavioral aspects.

However, our findings differed from a 2023 study, in which physicians were the primary source of GERD-related information for 30.9% of participants, suggesting greater involvement of healthcare professionals in patient education [17]. The limited exposure to healthcare-based education regarding GERD-related anxiety observed in our study indicates a potential opportunity for improving communication between healthcare providers and patients. Although lifestyle modifications are widely recommended, the 2024 Pakistan study evidence supporting specific nonpharmacological interventions targeting anxiety in GERD remains limited, and pharmacological acid suppression remains a central component of treatment [25]. Therefore, psychological interventions should be considered complementary approaches rather than replacements for established GERD therapies.

The bivariate analysis identified significant associations between previous professional diagnosis of GERD and age and marital status. Among participants diagnosed with GERD, most were older than 40 years and were married. In contrast, gender and educational level were not significantly associated with GERD symptoms. These findings are partially consistent with previous studies. A 2024 study conducted in Bahrain reported that GERD was significantly associated with marital status and certain lifestyle behaviors, including sleeping within one hour after dinner. The study found that most GERD patients were married, which closely corresponds with our finding among diagnosed participants [22].

Additionally, a 2024 Taiwanese population-based study reported significant sex differences in GERD-related probability patterns, suggesting that gender may influence GERD susceptibility more strongly than observed in our study population [13]. Differences in population characteristics, sample size, and lifestyle factors may explain variations between studies. In the multivariate logistic regression analysis, marital status remained the only independent sociodemographic characteristic significantly associated with GERD symptoms. Married participants demonstrated approximately 1.7-fold higher odds of GERD symptoms compared with single participants, whereas age was no longer statistically significant after adjustment. Similarly, a 2022 study reported a higher proportion of GERD among married individuals, although the association did not reach statistical significance [22]. These findings suggest that marital status may reflect differences in lifestyle patterns, dietary behaviors, or healthcare-seeking practices rather than representing a direct causal factor.

The present analysis identified significant self-reported associations between anxiety symptoms and both age and gender. Female participants represented a higher proportion among participants reporting anxiety symptoms compared with those without symptoms. Additionally, participants younger than 18 years showed a higher proportion of anxiety symptoms compared with older age groups.

These findings are consistent with previous evidence demonstrating sex differences in anxiety symptoms. A 2022 systematic review examining factors associated with gender differences in anxiety disorders confirmed that female participants consistently experience higher rates of anxiety disorders compared with male participants. The review reported that “the occurrence and comorbidity of anxiety disorders are significantly different between women and men, with research showing a greater impact on women” [26]. The higher proportion of anxiety symptoms among younger participants is also supported by global mental health data. According to the World Health Organization, approximately one in seven individuals aged 10-19 years experiences a mental health disorder, highlighting adolescence as a vulnerable period for psychological distress [27].

In the multivariate logistic regression analysis, female gender remained independently associated with anxiety symptoms, with female participants showing higher odds of reporting anxiety compared with male participants. Participants younger than 18 years had higher odds compared with older age groups; however, this finding should be interpreted with caution because only 10 participants (2.6%) were younger than 18 years.

This study has some limitations. Its cross-sectional design precluded establishing relationships between anxiety and GERD. In addition, convenience sampling and the use of an online self-administered questionnaire may have introduced selection and response bias, while self-reported data may have been subject to recall or reporting bias. Because the study was conducted predominantly among adults residing in Jeddah, with a small number of participants younger than 18 years, the findings may not be generalizable to other regions or age groups in Saudi Arabia. In addition, the absence of formal psychometric validation of the anxiety assessment limits the interpretation of the reported anxiety symptoms. Another limitation is the inclusion of a small number of participants younger than 18 years (10 participants, 2.6% of the total sample). Given the small size of this subgroup, age-related findings involving participants younger than 18 years should be interpreted cautiously. Finally, the predominance of female participants may have influenced the proportion estimates and reduced the representativeness of the study population.

## Conclusions

This study identified a high frequency of self-reported anxiety symptoms and reflux-related symptoms among the surveyed participants in Jeddah. A substantial proportion of participants also perceived that stress, or anxiety was associated with worsening of their reflux-related symptoms. However, these findings represent self-reported associations and do not establish a clinical diagnosis of anxiety-associated GERD or a causal relationship between anxiety and GERD. Although general awareness of this association was relatively good, important knowledge gaps remained.

The findings also identified significant sociodemographic differences in GERD and anxiety symptoms, underscoring the need for integrated healthcare approaches that address both gastrointestinal and psychological factors. Strengthening public education and increasing the involvement of healthcare professionals may improve awareness, early recognition, and the comprehensive management of GERD and anxiety.

## Appendices

Questionnaire used in the study

Section I. Assessment of Sociodemographic Characteristics

1. Age

□ <18 years

□ 18-40 years

□ >40 years

2. Gender

□ Male

□ Female

3. Marital status

□ Single

□ Married

4. Educational level

□ Primary school

□ Secondary school

□ University

□ Postgraduate studies

Section II. Evaluation of the Prevalence of Anxiety and GERD-Related Symptoms

1. Do you regularly experience symptoms of anxiety?

□ Yes

□ No

2. Have you ever been diagnosed with GERD by a healthcare professional?

□ Yes

□ No

3. What GERD symptoms do you experience?

□ Heartburn

□ Sour taste in the mouth

□ Difficulty swallowing

□ Chronic cough

□ Other (please specify): __________________

4. How often do you experience anxiety symptoms?

□ Daily

□ Weekly

□ Monthly

□ Rarely

□ Never

5. Do you use any medication that improves GERD symptoms?

□ Yes

□ No

If yes, please specify: __________________

Section III. Assessment of Public Awareness Regarding the Relationship Between GERD and Mental Health

1. Have you heard about the impact of GERD on mental health?

□ Yes

□ No

2. Do you think GERD can affect mental health?

□ Yes

□ No

□ I don't know

3. Are you aware that GERD may have psychological as well as physical effects?

□ Yes

□ No

□ I don't know

4. What is your main source of information about GERD?

□ Healthcare professionals

□ Social media

□ Family

□ Friends

□ Personal research

Section IV. Determination of the Association Between Anxiety and GERD

1. Do you notice an increase in GERD symptoms when feeling anxious or stressed?

□ Yes

□ Sometimes

□ No

2. How would you describe the relationship between anxiety and GERD based on your personal experience?

□ Strong

□ Moderate

□ Weak

□ No relationship

□ Unsure

3. Are there specific situations in which anxiety worsens your GERD symptoms?

□ Yes (please specify): __________________

□ No

Section V. Effects of GERD on Lifestyle

1. How much does GERD impact your overall quality of life?

□ Affects

□ Does not affect

□ I did not notice

2. Has GERD affected your sleep patterns?

□ Affects

□ Does not affect

□ I did not notice

3. Has GERD affected your dietary habits (e.g., avoiding certain foods)?

□ Affects

□ Does not affect

□ I did not notice

4. Do you exercise regularly?

□ Yes

□ No

5. Do you notice that environmental factors (such as odours or noise) affect your GERD symptoms?

□ Yes

□ No

Section VI. Assessment of Knowledge and Management Practices Regarding GERD-Related Anxiety

1. How do you manage anxiety related to gastroesophageal reflux? (More than one answer may be selected.)

□ Medical consultation

□ Relaxation techniques

□ Regular exercise

□ Other (please specify): __________________

2. Have you received information from healthcare providers on ways to reduce GERD-related anxiety?

□ Yes

□ No

3. On a scale of 1 to 5, how confident are you in managing anxiety symptoms related to GERD?

□ 1 = Very low

□ 2 = Low

□ 3 = Moderate

□ 4 = High

□ 5 = Very high

4. Are you open to learning more about the link between GERD and anxiety through educational resources or support groups?

□ Yes

□ No
